# Multi-Ship Control and Collision Avoidance Using MPC and RBF-Based Trajectory Predictions

**DOI:** 10.3390/s21216959

**Published:** 2021-10-20

**Authors:** Myron Papadimitrakis, Marios Stogiannos, Haralambos Sarimveis, Alex Alexandridis

**Affiliations:** 1 Department of Electrical and Electronic Engineering, University of West Attica, 12241 Aigaleo, Greece; m.papadimitrakis@uniwa.gr (M.P.); mstogia@uniwa.gr (M.S.); 2 School of Chemical Engineering, National Technical University of Athens, 15780 Zografou, Greece; hsarimv@central.ntua.gr

**Keywords:** autonomous vessels, collision avoidance, model predictive control, radial basis function networks, trajectory optimization

## Abstract

The field of automatic collision avoidance for surface vessels has been an active field of research in recent years, aiming for the decision support of officers in conventional vessels, or for the creation of autonomous vessel controllers. In this paper, the multi-ship control problem is addressed using a model predictive controller (MPC) that makes use of obstacle ship trajectory prediction models built on the RBF framework and is trained on real AIS data sourced from an open-source database. The usage of such sophisticated trajectory prediction models enables the controller to correctly infer the existence of a collision risk and apply evasive control actions in a timely manner, thus accounting for the slow dynamics of a large vessel, such as container ships, and enhancing the cooperation between controlled vessels. The proposed method is evaluated on a real-life case from the Miami port area, and its generated trajectories are assessed in terms of safety, economy, and COLREG compliance by comparison with an identical MPC controller utilizing straight-line predictions for the obstacle vessel.

## 1. Introduction

In the last two decades, research on automatic collision avoidance and optimal path planning for surface vessels has intensified, driven by the ever-growing density of maritime traffic in narrow waterways, such as gulfs, ports, and canals [1]. Motivated by the design of autonomous surface vehicles (ASV) controllers, but also aiming for the decision support of officers on watch in conventional vessels [2], control and optimization tools that ensure the safety and the cost effectiveness of navigational actions are being intensively developed. These tools are perceptive of the surrounding environment through arrays of sensors, radars, and other positioning and communication aids. In this context, the automatic identification system (AIS) encompasses most of the aforementioned technologies in order to gather positioning and other vessel data. The already vast AIS comprises an ever-expanding worldwide maritime trajectory dataset, which is made available by vessels, port authorities, and other platforms in charge of efficient and safe maritime path planning. Given the fact that the majority of vessel accidents are related to erroneous handling rather than equipment failure or environmental conditions [3], these tools aim to phase out the human officers on watch as vessel controllers, or at least augment their navigational decision-making using optimization- and prediction-based methods.

The formulation of the trajectory optimization problem used in collision avoidance controllers must take multiple aspects of vessel navigation into account, while being perceptive of their surrounding environment in real time. The generation of control actions that will result in a trajectory remaining sufficiently clear from any stationary or moving objects is not the sole objective; an efficient controller should also ensure the economy of the control actions, as well as the adherence to the collision avoidance rules, commonly known as the COLREGs [4]. Multiple collision avoidance controllers have been proposed that fulfil the aforementioned specifications; in [5], a hierarchical multiobjective optimization problem is formulated, which generates an intermediate waypoint for the controlled vessel while accounting for the good seamanship rules. In [6], a fuzzy-Bayesian collision avoidance controller is formulated capable of addressing multiple obstacle vessels at once. In [7], optimal trajectories for the collision avoidance problem are generated using a B-Spline-based search algorithm. Lastly, in [8], a collision avoidance controller utilizes a probabilistic method in order to infer the one-step-ahead position of obstacle vessels, while also accounting for non-COLREG-compliant obstacle vessels.

In general, it has been observed that controllers that are not model-based can have trouble incorporating crucial aspects of the trajectory optimization problem, thus compromising practicality. Without a working model of the controlled vessel, its maneuvering capabilities cannot be easily included in the formulation, and neither can the effect of environmental conditions be quantified [9,10]. For these reasons, model predictive control (MPC) emerges as an effective control method for the problem at hand because it utilizes a model of the plant in order to compute an optimal control trajectory based on the predicted trajectory of other ships in the vicinity. As a framework, MPC can account for the uncertainties of both the utilized model of the plant and the trajectory prediction models of other ships, while also incorporating all possible control objectives (such as navigational risk, course smoothness, or deviation from the original path) in a single cost function. Some collision avoidance controllers based on MPC have been proposed in the literature; a robust MPC controller utilizing straight-line obstacle vessel trajectory predictions is proposed in [9], capable of COLREG compliance and handling of multiple obstacles. In [11], motion planning for an autonomous vessel using a sampling-based MPC method takes place. In [12], an MPC controller for the collision avoidance task is built by approximating the behavior of an LQR controller, thus ensuring asymptotic stability of the system. In [13], a neural network used to approximate the MPC response for the generation of COLREG compliant trajectories for multi ship encounters is presented. In addition, MPC has been integrated in distributed control frameworks of multi-ship schemes; for example, a distributed MPC scheme has been employed for a multi-vessel formation controller with collision avoidance capabilities [14], or for the robust distributed control of multiple vessels operating for the inter-terminal transport of containers [15].

It becomes apparent that for the scope of the collision avoidance task, information about the future trajectories of other ships plays a central role. Prevalent in non-data driven methodologies already used for the vessel trajectory prediction (VTP) problem is the first principles-based modeling technique [16], carrying a number of significant shortcomings, such as their inherent complexity, which has a greater negative impact due to the fact that the model is usually employed multiple times within the duration of each MPC sample. In order to simplify the solution of the employed kinematic differential equations and facilitate the real-time prediction of future states, these types of models are usually created using several assumptions that try to approximate real-world conditions, but also make the final model far less accurate. Therefore, one should employ a more sophisticated, data-driven approach for the creation of effective trajectory prediction models that are included in MPC controllers. Machine learning has answered the call of producing highly accurate models, which may be easily integrated in predictive frameworks through the use of black-box modeling, and more specifically, artificial neural network (NN) approaches [17]. NNs employ different architectures in order to remap the original non-linear problem to a higher-dimensional input space and approximate its dynamics utilizing standard functions. In this context, various NN techniques have been successfully utilized in control frameworks solving the vessel trajectory prediction problem.

Feedforward NN architectures, most commonly represented by the multilayer perceptrons (MLPs), have been employed to solve the vessel trajectory prediction problem as in [18,19], where MLP NNs are trained using the well-established backpropagation algorithm outperforming rival methodologies, i.e., linear models and Kalman filters. In [19], a real AIS dataset gathered from the confined space of a river waterway is used to approximate the vessel dynamics in such environments. Backpropagation has been the baseline of more efficient training methods as in [20], where different computational intelligence approaches like differential evolution, genetic algorithms, and swarm-based techniques are used to modify the original backpropagation algorithm in order to create more accurate feedforward NN models. Other NN architectures, like support vector machines (SVMs), have been employed in conjunction with computational intelligence optimization techniques, i.e., the particle swarm optimization (PSO) algorithm, on AIS datasets to solve the vessel trajectory prediction problem [21]. In most cases, the inherent abilities of NN architectures that can meet the standard of high accuracy are limited to a one-step ahead prediction horizon, in the sense that multi-step ahead predictions would require an approximation of unknown future states to be made and present an error enlarged through propagation to the end of the prediction horizon. Such an error would become critically high after a small number of steps, rendering the control framework useless.

To overcome this problem, long-term trajectory prediction approaches have been devised with the inclusion of memory features, such as the recurrent neural networks (RNNs), with their most notable representative, i.e., the long short-term memory (LSTM) NNs already used in the context of the vessel trajectory prediction problem [22,23,24,25]. Besides trajectory modeling and prediction in open waters, advances have also been made in crowded port waters as in [26], where another modification of the RNNs, namely the bidirectional gated recurrent unit, is used to address the vessel trajectory prediction problem, outperforming standard NN methods in such scenarios. Gated recurrent units are promising candidates for predicting the collective behavior of vessel fleets [27].

Radial basis function (RBF) networks form a unique NN architecture belonging to the general feedforward NN category. RBFs differ from other NN architectures, having simpler structures, employing faster training algorithms, and usually producing more accurate models than MLPs. Within the context of vessel trajectory prediction, RBFs have been integrated in control frameworks by approximating unknown vessel parameters [28,29,30]. Recently, RBFs have been applied on real AIS data in order to produce highly accurate models for one-step and multi-step ahead predictions [31], showing their potential in being integrated to receding horizon control methodologies.

Remarkably, in the collision avoidance research literature, there are no instances where the multi-step-ahead trajectory prediction of moving obstacles is addressed in such a systematic manner; usually these trajectories are either known a priori, or there are no obstacle ships present whatsoever. An exception occurs in [9], where straight line trajectory predictions are employed, based on estimates of current course and speed for the moving obstacle. In this work, a multi-ship MPC controller utilizing RBF obstacle trajectory prediction models trained using real AIS data is presented for the collision avoidance task. The main contributions of this work are as follows: first, we introduce a novel MPC scheme for collision avoidance control, where nonlinear data-driven models are used to predict the trajectories of obstacle ships; to the authors’ best knowledge, this is the first such instance in the literature. The previous state-of-the-art approach of using straight-line obstacle trajectory predictions may have yielded satisfactory approximation results in open sea case studies, where ships are expected to travel in a straight line, but is of limited practical use for the cases of narrow gulfs, ports, or canals, where ships are expected to maneuver while navigating through, thus resulting to nonlinear trajectories. Secondly, the aforementioned nonlinear models are trained using an AIS data-driven methodology, which, once again, constitutes an original approach in the context of vessel collision avoidance. Using real trajectories as training data increases the practicality of the proposed scheme, since the resulting trajectory prediction models capture the dynamics of real vessels. The proposed method is tested in a collision avoidance case study at the Miami port area, and its performance is illustrated by the comparison with an MPC controller employing straight-line obstacle prediction models, which corresponds to the current state-of-the-art approach [9].

The paper is structured as follows. In Section 2, the AIS-data-driven methodology for the creation of the RBF trajectory prediction models is presented. In Section 3, some preliminaries on maritime collision avoidance and optimal trajectory generation are described, and later, the proposed method is presented. In Section 4, the case study based on the port of Miami is outlined, and the simulation results are discussed in depth. Lastly, in Section 5, concluding remarks are made.

## 2. AIS-Data-Based Trajectory Prediction Models

### 2.1. Radial Basis Function Neural Networks

RBF NNs have been successfully employed to approximate nonlinear system dynamics in order to predict future system states in numerous diverse applications [32,33]. Their success can be mainly attributed to their structure, which is simpler when compared to other NN architectures, as they comprise a single hidden layer, attached linearly to the network output. This property not only allows for using very fast training algorithms, but also makes RBF NNs suitable for integration in MPC schemes, as (a) it facilitates the controller design [34], and (b) helps to solve the MPC online optimization problem in shorter computational times [35], thus rendering such schemes applicable to systems with fast dynamics [36]. Another property that makes RBF NNs a popular modeling method in predictive control schemes is related to their increased approximation capabilities [37,38]. Indeed, notwithstanding their simple structure, RBF NNs have proven to be more accurate in modeling various nonlinear systems when compared to other machine learning methods, including MLPs, support vector machines (SVMs), random forests, etc. [39]. Especially within the context of the vessel modeling and control problems, RBFs have already shown great potential in modeling unknown vessel parameters [28,29], in order to create models capable to be integrated in trajectory tracking control algorithms. Furthermore, in a recent publication [31], it has been shown that RBF NNs trained with the fuzzy means (FM) algorithm outperform other data-driven techniques such as MLPs when modeling vessel trajectories based on AIS data.

Training an RBF NN is a process consisting mainly of two phases. The first phase is performed by applying a clustering technique on the training dataset in order to identify the centerpoint and optimized parameters of a number of radially symmetric basis functions called nodes. The incorporation of radial basis functions (e.g., Gaussian, quadratic, etc.) is the first main difference between RBFs and other feedforward NNs. The linear combination of these nodes produces the output prediction of the network. Finding the node weights is the goal of the second phase, a problem trivially solved by least squares.

**Figure 1:**
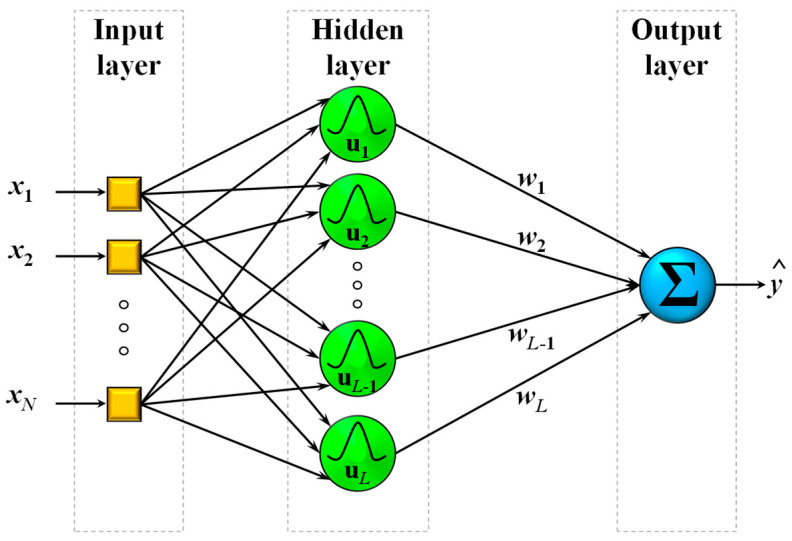
A typical radial basis function network structure using Gaussian RBFs.

A typical RBF network can be seen in Figure 1. The structure comprises three distinct layers, the first of which is the input layer and has the sole purpose of distributing the *N* inputs to the *L* nodes of the hidden layer. The second point differentiating RBFs from other architectures is the existence of only one hidden layer of *N*-dimensional nodes. In order to produce a prediction $\hat{y}\left(k\right)$ given an input datapoint x(k), at first the Euclidean norm is used to calculate the activity $\mu_{l}\left(x\left(k\right)\right)$ for every node l by using the difference between the *k*-th input vector x(k) and the *l*-th node center ${\hat{u}}_{l}$, such that

(1) $$\mu_{l}\left(x(k)\right)=\|x(k)-{\hat{u}}_{l}\|=\sqrt{\sum_{i=1}^{N}{\left(x_{i}(k)-{\hat{u}}_{i,l}\right)}^{2}},k=1,\ldots ,K$$

The activity acts as input to the free parameters of each node according to the chosen RBF. The hidden node response vector z(k) for the *k*-th datapoint is given by

(2) $$z\left(k\right)=\left[g\left(\mu_{1}\left(x\left(k\right)\right)\right),g\left(\mu_{2}\left(x\left(k\right)\right)\right),...,g\left(\mu_{L}\left(x\left(k\right)\right)\right)\right]$$

where *g* corresponds to the chosen activation function. Note that in this work, a thin plate spline function is employed

(3) $$g\left(\mu_{l}\left(x\left(k\right)\right)\right)=\mu_{l}^{2}\left(x\left(k\right)\right)\cdot \log\mu_{l}\left(x\left(k\right)\right)$$

due to the fact that it is an established choice as an RBF kernel producing models of high accuracy [40], but also because there are no tunable parameters other than the actual input to the function. Such parameters would require optimization techniques to be included in the training process, e.g., employment of the Gaussian function would need kernel width optimization, which is usually performed by cpu-intensive iterative algorithms or suboptimal trial-and-error techniques.

Finally, the network’s prediction is calculated by linearly combining the hidden note responses such that

(4) $$\hat{y}\left(k\right)=z\left(k\right)\cdot w$$

where **w** is a vector containing the node weights.

### 2.2. The Fuzzy Means (Fm) Training Algorithm

For a given training dataset where **Y** denotes the real outputs, and after the hidden node responses **Z** are formulated, the weight vector **w** can be trivially calculated by least squares in matrix form

(5) $$w^{T}=Y^{T}\cdot Z\cdot {\left(Z^{T}\cdot Z\right)}^{-1}$$

thus completing the second training phase in one easy step.

The first phase of training requires a clustering algorithm to be applied to the training dataset, in which case the fuzzy means (FM) algorithm is a great candidate for this task [39]. Following the notation of the previous example, let us suppose a system with *N* normalized input variables $x_{i}$. At first, each input variable domain must be partitioned into *s* 1-D fuzzy sets (FS). Each fuzzy subspace $A^{l}$, where $l=1,2,\ldots ,s^{N}$, is formed by the selected $s^{N}$ fuzzy sets according to the respective input variable. This process creates a *N*-dimensional grid, where all intersection points, also called nodes, are candidates to become RBF centerpoints. The FM algorithm undertakes the task of selecting the appropriate candidate nodes that will be assigned as RBF centers. To perform the selection procedure, a membership function

(6) $$\mu_{A^{l}}\left(x\left(k\right)\right)=\left\{ \begin{array}{ll} 1-d_{r}^{l}\left(x\left(k\right)\right), & if\;d_{r}^{l}\left(x\left(k\right)\right)\leq 1\\ 0, & if\;otherwise \end{array}\right.$$

determines whether an input vector lies within the domain of an RBF centered around a candidate node. In the simple case where all input variable spaces are equally partitioned, the following distance metric can be used to assign *N*-dimensional spherical domains to each candidate node

(7) $$d_{r}^{l}\left(x\left(k\right)\right)=\sqrt{\sum_{i=1}^{N}{\left(a_{i,ji}^{l}-x_{i}\left(k\right)\right)}^{2}}/\sqrt{N}\delta a$$

where x(k) is the *k*-th input vector, $a_{i,ji}^{l}$ is the centerpoint of fuzzy subspace $A^{l}$, and δa is the sphere radius, which is the same for each input. The FM algorithm uses a fast non-iterative procedure to find a subset of the subspaces, so that the final RBF NN’s hidden layer comprises only the fuzzy sets, which sufficiently cover all training datapoints, in the sense that each datapoint is included in at least one fuzzy set. For an in-depth description of the FM algorithm, the reader may refer to [39].

### 2.3. Data Preprocessing

Best modeling practices mandate that a training dataset should be error- and noise-free, a case that is far from truth when using data from AIS transceivers [41,42,43,44,45]. AIS data are irregularly sampled and contain heavy noise, missing data, and erroneous values. Thus, before employing any modeling technique, rigorous preprocessing is in order.

The Marine Cadastre service (www.marinecadastre.gov, accessed on 25 July 2021) has been the source of all data used in this work. MarineCadastre.gov is a is a service that gathers and publicly provides AIS data to marine planning initiatives. In this work, data from all days between 1 July 2019 and 30 June 2020 have been included and filtered to keep vessels sailing an area around the port of Miami covered by the geolocation rectangle defined by the latitudes of 25.720° through 25.840° and the longitudes of −80.145° through −80.042°. To conform to the initial assumption of similar size and similar dynamics, we allowed only cargo ships sailing on engine power into the dataset, further filtering the dataset to yield a total of 180 vessels.

To address the problems of sample irregularity, noise, and erroneous values, the dataset was resampled to 120 s, which was deemed enough to capture the high inertia dynamics of large cargo ships. The interpolation technique applied on the data to perform the resampling was the Akima piecewise cubic interpolation [46], which is quite effective on geolocation data, performing a mild denoising as well. A heuristic that rejects very far-off outlier values due to GPS errors was also applied. The trajectories were split in data samples, with each one containing ten consecutive vessel positions. Note that each trajectory’s starting point should be the last point of the previous one resulting in an overlap of one point, but this final position will be used as the model’s output, so no actual overlapping exists within the input data. The resampling and splitting process yielded a total of about 14 k samples from 3.1 k resampled trajectories of the initial 180 vessels. Algorithm 1 depicts the step-by-step procedure of preprocessing.
**Algorithm 1** Preprocessing Stage
1: Process each entry in the common dataset so that it contains only the following: Vessel ID, timestamp, latitude, and longitude. Reject all other information.2: Sort dataset by vessel ID and sort each vessel data by date.3: Apply resampling and outlier filtering on the data of each vessel to achieve a resampling of 120 s.4: Split vessel data into trajectories containing ten consecutive vessel positions each.5: Create final preprocessed dataset, which should contain the vessel ID and final 10-position trajectories.

### 2.4. Modeling Procedures

AIS transceiver equipped vessels are able to record and exchange timestamped information, including geolocation, speed, direction, vessel identification, and specifications data. Vessel trajectory prediction algorithms integrated with collision avoidance techniques can incorporate trajectory prediction models in order to identify imminent threats and navigate safely and efficiently within heavily crowded port areas or open seas.

Let us suppose an available AIS dataset, comprising an arbitrary number of $T^{v}$ trajectories for a total of *V* vessels, where v=1,2,…,V. Let us also suppose that the included trajectories contain an arbitrary number of $K^{v,t}$ AIS messages $AISm_{k}^{v,t}$ (timestamped geolocation and other data). In this work, for simplicity reasons, we employ the following format in AIS messages

(8) $$AISm_{k}^{v,t}=\left\{\begin{array}{ccc} Ts_{k}^{v,t} & y_{k}^{v,t} & x_{k}^{v,t} \end{array}\right\}$$

where $k=1,2,\ldots ,K^{v,t}$, and $Ts_{k}^{v,t}$ denotes the message timestamp, while $y_{k}^{v,t}$ and $x_{k}^{v,t}$ are the respective latitude and longitude contained in the *k*-th AIS message for the *t*-th trajectory of the *v*-th vessel. The fact that there are unknown parameters, e.g., the state and controls of the vessels, prohibits the use of kinematics in calculating future vessel states. Nevertheless, the vessel dynamics exist in the information hidden within the dataset and can be extracted and, in most cases, approximated by using a black-box modeling technique such as RBF NNs. We can assume that a common underlying pattern exists in the dynamics of same-size vessels executing similar maneuvers, for example, when approaching or leaving a port, when berthing, when crossing waterway paths, etc. Thus, if a suitable dataset of sufficient size is made available, an RBF NN can be trained to perform one-step-ahead predictions about a vessel’s future geolocation by using past AIS messages as seen in the following equations

(9) $$\left\{\begin{array}{c} \Delta {\hat{y}}_{k+1}^{v,t}\\ \Delta {\hat{x}}_{k+1}^{v,t} \end{array}\right\}=RBF^{}\;NN\left(\begin{array}{ccc} AISm_{k}^{v,t} & \ldots & AISm_{k-N}^{v,t} \end{array}\right)$$

(10) $$\begin{array}{l} {\hat{y}}_{k+1}^{v,t}=y_{k}^{v,t}+\Delta {\hat{y}}_{k+1}^{v,t}\\ {\hat{x}}_{k+1}^{v,t}=x_{k}^{v,t}+\Delta {\hat{x}}_{k+1}^{v,t} \end{array}$$

where *N* is the number of past AIS messages given as inputs to the RBF NN.

The accuracy and simple structures of FM-trained RBF NNs make them ideal to be integrated in multi-step-ahead predictive control formulations. Receding horizon techniques require models of very high accuracy due to the inevitable error enlargement through propagation. This effect appears when the incorporated model is expected to recurrently make future predictions based on its own previous output throughout the prediction horizon. The problem is further worsened with the increase of the prediction horizon and can ultimately drive the control algorithm to failure. Another important point to be noted is that the linear combination used to produce an RBF NN’s prediction is a simple and very fast calculation, a fact that benefits model predictive control (MPC) frameworks [36], which require an optimization problem to be solved iteratively and expect a significant number of model predictions to be made in order to converge to the optimal solution at each timestep.

Delta values of the last position of each sample were used as the model’s output, while the first nine positions were the model’s input

(11) $$\left\{\begin{array}{c} \Delta {\hat{y}}_{k+1}^{v,t}\\ \Delta {\hat{x}}_{k+1}^{v,t} \end{array}\right\}=RBF\;NN\left(\begin{array}{cc} y_{k}^{v,t} & \begin{array}{cc} x_{k}^{v,t} & \begin{array}{ccc} \ldots & y_{k-8}^{v,t} & x_{k-8}^{v,t} \end{array} \end{array} \end{array}\right)$$

The $\Delta {\hat{y}}_{k+1}^{v,t}$ and $\Delta {\hat{x}}_{k+1}^{v,t}$ values may be added to the last input position to calculate the final predicted vessel position. Based on the above procedure, the results of the modeling process produced an RBF model of very high accuracy [31]. The step-by-step procedure of the modeling stage can be seen in Algorithm 2.

Note that the number of past inputs was determined after a trial-and-error procedure, where several RBF models were trained using a different number of inputs. After testing inputs in the range of 3 to 15 past vessel positions, data obtained on model performance showed that using less than nine inputs produced models with reduced prediction accuracy, while using more than nine inputs increased the model’s complexity without any significant accuracy gain compared to the model using nine inputs.
**Algorithm 2** Modeling Stage
1: Load final preprocessed dataset.$2:\;\mathrm{Replace}\;\mathrm{the}\;\mathrm{final}\;\mathrm{value}\;\mathrm{of}\;\mathrm{all}\;\mathrm{included}\;10-\mathrm{position}\;\mathrm{trajectories}\;\mathrm{with}\;\mathrm{the}\;\mathrm{respective}\;\mathrm{delta}\;\mathrm{value}\;\mathrm{according}\;\mathrm{to}\;\left\{\begin{array}{c} \Delta y_{10}^{v,t}\\ \Delta x_{10}^{v,t} \end{array}\right\}=\left\{\begin{array}{c} y_{10}^{v,t}-y_{9}^{v,t}\\ x_{10}^{v,t}-x_{9}^{v,t} \end{array}\right\}$, so that each trajectory sample is in the form $\left[\begin{array}{ccccccccc} y_{1}^{v,t} & x_{1}^{v,t} & y_{2}^{v,t} & x_{2}^{v,t} & \ldots & y_{9}^{v,t} & x_{9}^{v,t} & \Delta y_{}^{v,t} & \Delta x_{}^{v,t} \end{array}\right]$.3: Randomly permute the trajectory samples of each vessel.4: Split the trajectory samples of each vessel into training, validation, and testing subsets (in this work a 50%–5%–25% percentage split is used). Do this so that all vessels contribute to all three subsets according to the chosen splitting.5: Merge all subset samples, e.g., all training samples of all vessels together in one single dataset that will be used for training. Do the same for the validation and testing subsets.6: Normalize the inputs and outputs of the training subset. Apply the normalization coefficients to the validation and testing subsets.$7:\;\mathrm{Apply}\;\mathrm{the}\;\mathrm{fuzzy}\;\mathrm{means}\;\mathrm{algorithm}\;\mathrm{on}\;\mathrm{the}\;\mathrm{training}\;\mathrm{and}\;\mathrm{validation}\;\mathrm{dataset}\;\mathrm{using}\;\mathrm{the}\;\mathrm{nine}\;\mathrm{first}\;\mathrm{sets}\;\mathrm{of}\;y_{}^{v,t}$ − $x_{}^{v,t}$ values as inputs and the last set of $\Delta y_{}^{v,t}$ − $\Delta x_{}^{v,t}$ values as output.8: Final model is in the form of Equation (11).

Moreover, a series of tests has been performed by the recurrent application of this model based on a horizon of 5 timesteps for all trajectories of the testing subset, where, at each successive timestep, the model had to use an increasing number of its own previous predictions. As the model uses more of its past predictions, accuracy decreases due to the enlargement of the propagated prediction error. Such a test can provide intuition on the models’ ability to be incorporated in receding horizon predictive frameworks. The quality metrics used for these tests were the root mean squared error (RMSE) and the root mean squared haversine formula distance (RMSHFD). The haversine formula is commonly used to measure great circle distances on spherical surfaces. Table 1 presents the performance metrics obtained after the recurrent application of the chosen model in order to make predictions for the full length of the trajectories included in the testing subset of the training procedure. Mean RMSE values for the two outputs of the model, namely the latitude and longitude, are provided in degrees, wherein can be seen that the error lies in the order of 1.5 thousandth of a degree. The mean RMSHFD metric shows the respective error margin in meters when combining the two model outputs to get the actual predicted future vessel position for all tested trajectories. More details on the modeling procedure for the one-step ahead models, including detailed results and comparison with other machine learning approaches, can be found in [31].

## 3. MPC for Multi-Ship Collision Avoidance

### 3.1. Preliminaries on Maritime Collision Avoidance and Trajectory Generation

**Figure 2:**
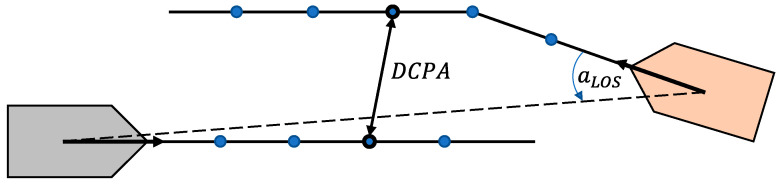
Illustration of the CPA metrics, as well as the LOS angle concept.

The objective of maritime collision avoidance is the generation of a risk-free trajectory that the controlled vessel should follow. A well-defined and effective method of assessing collision risk in the near future is the closest point of approach (CPA). Stemming from the concept of the CPA, two metrics are defined: time to CPA (TCPA) and distance to CPA (DCPA) (see Figure 2). A discussion regarding the quick calculation of TCPA and DCPA using the line-of-sight (LOS) distance between the controlled vessel and the obstacle ship is presented in [5]. These metrics depict the urgency of the collision danger of vessel *i* with another vessel *j* as well as its magnitude, and by specifying lowest acceptable thresholds $d_{min}$ and $t_{min}$ concerning the minimum DCPA and minimum TCPA, respectively, one can construct a risk cost function, as presented in [5].

(12) $$f_{r,ij}=\{\begin{array}{c} \exp\left(a_{0}\left(d_{min}-DCPA\left(T_{i},T_{j}\right)+t_{min}-TCPA\left(T_{i},T_{j}\right)\right)\right)-1,\;if\;\;\;\\ \;DCPA\left(T_{i},T_{j}\right)\leq d_{min}\;\;\;and\;\;\;TCPA\left(T_{i},T_{j}\right)\leq t_{min}\\ 0,\;\;\;if\;otherwise \end{array}$$

Here, $a_{0}$ is a scaling parameter, and $T_{i}$ denotes the trajectory matrix containing the x-y position of the *i* vessel for every timestep

(13) $$T_{i}=\left[\begin{array}{cc} x_{1} & y_{1}\\ \vdots & \vdots \\ x_{n} & y_{n} \end{array}\right].$$

By combining TCPA and DCPA, the spatial-temporal nature of a maritime collision risk with vessel *i* is successfully reflected. The physical interpretation of Equation (12) is that a candidate trajectory with larger minimum distance from an obstacle ship occurring at an earlier time will always be safer than a path with a smaller minimum distance and/or earlier time of occurrence. Common values for $t_{min}$ and $d_{min}$ are 10 min and 0.6 nm; because the present paper is concerned with collision avoidance in busy waterways such as ports, a lower $d_{min}$ value of 0.4 nm is used. In any case, Equation (12) can be readily incorporated in the cost function of an MPC optimization problem formulation.

A second item in the domain of trajectory generation is efficiency. Vessels should strive to not deviate too much from their original course when addressing a collision risk with another vessel. The efficiency of the generated trajectory $T_{i}$ for vessel *i* can be reflected by calculating the sum of absolute deviations from the original trajectory $T_{OG,\;i}$

(14) $$f_{d,i}=\|T_{OG,i}-T_{i}\|.$$

**Figure 3:**
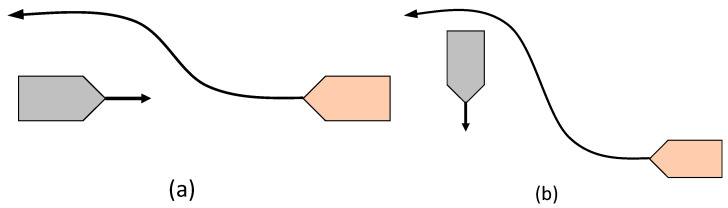
(**a**) A head-on situation between two ships; (**b**) a crossing situation between two ships (give-way); the orange ship must give way to the crossing ship on its starboard side.

Next, an important requirement to be fulfilled when addressing the problem of collision avoidance are the COLREGs [4]. The implementation of the COLREGs restricts the domain of possible candidate paths according to the type of encounter, for example ‘head-on,’ ‘crossing,’ and ‘overtaking.’ Head-on vessels should pass each other on the port side, while a vessel crossing from the starboard side should be given the right of way. A visual depiction of the encounter rules takes place in Figure 3. Multiple approaches for the modeling of the COLREG rules have been made in the literature [2,5,8], although these are usually concerned with a one-step-ahead calculation. However, for the case of an MPC controller, in order to ensure COLREG compliance for a candidate trajectory, all of its waypoints must be taken into account. By assuming that the LOS angle is increasing in the anti-clockwise direction, one needs to evaluate whether the LOS angles of each sequential trajectory timestep position are increasing monotonically, in order to confirm the compliance of the trajectory for the ‘head-on’ and ‘give-way’ situations.

**Figure 4:**
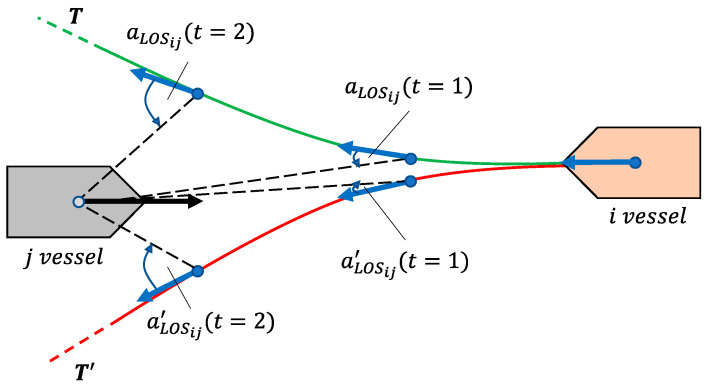
Head-on situation between vessels *i* and *j*; the LOS angle can be used to assess the COLREG compliance of a candidate trajectory.

The idea is depicted in Figure 4, where a head-on encounter between vessels *i* and *j* occurs; here, the LOS angles for trajectory $T_{i}$ monotonically increase, and therefore, it is deemed as compliant. In contrast, the monotonically decreasing LOS angles of the $T_{i}^{\prime }$ trajectory confirm its non-compliance as per the COLREGs intentions. A penalty for non-compliance of a vessel *i* encountering a vessel *j* in a ‘head-on’ or ‘give-way’ situation can therefore be formulated,

(15) $$P_{ij}=\{\begin{array}{c} 1,\;\;if\;a_{LOS_{ij}}↘\;\;\\ 0,\;\;if\;otherwise \end{array},$$

where $a_{LOS_{ij}}$ is the LOS angle vector, calculated for each trajectory point of encountering vessel *i* and the current position of encountered vessel *j*.

Next, the generated vessel trajectory, apart from being safe and COLREG compliant, should also take into account the maneuvering capabilities of the controlled vessel, i.e., it should be guaranteed that the trajectory is technically possible to be tracked by the vessel. The feasible search domain of the trajectory optimization problem can be constructed by a purely geometric approach in the case of a one-step-ahead calculation, such as in [5], where the design variables are the vessel’s next position and course. However, the extension of this geometric approach to multiple-steps-ahead requires the application of nonlinear constraints that would bound every sequential vessel position with its previous one, in order to enforce technical feasibility. For this reason, a model-based approach is preferred. The Nomoto models constitute a class of vessel course models that are tailored for this task, and have been widely adopted, not only for the design of collision avoidance schemes [47], but also for path tracking controllers [48]. The 1st order linear Nomoto model is shown as follows:

(16) $$T_{s}\frac{d\omega }{dt}+\omega =K_{s}a.$$

Here, *ω* is the angular velocity of the vessel, while a is the control input to the vessel’s rudder. The maneuvering capabilities of the vessel are reflected by the $T_{s}$ and $K_{s}$ constants, called time constants and rudder gain constants, respectively, while typical values are in the [0.5, 2] range for both. Solving the differential Equation (16) by assuming constant rudder angle input for a *t* time interval, the 1st order linear Nomoto model can be discretized as follows [8]:

(17) $$\Delta \theta \left(t\right)=K_{s}\;a\left(t-T_{s}+T_{s}\;\exp\left(\frac{t}{T_{s}}\right)\right)$$

Here, Δ*θ* is the course change that would occur if a control input of a was applied and held for a time period of t. By setting this time period *t* as the discretization interval Δt, a course model can be used to create a discrete vessel position model as follows

(18) $$\theta_{k+1}=\theta_{k-1}+\Delta \theta_{k}\left(a_{k}\right)\;x_{k+1}=x_{k}+\cos\left(\theta_{k+1}\right)V_{k}\;\Delta t\;y_{k+1}=y_{k}+\sin\left(\theta_{k+1}\right)V_{k}\;\Delta t$$

Here, $\theta_{k}$, $x_{k}$, $y_{k}$ is the current course, horizontal displacement, and vertical displacement according to a global reference frame, respectively, while $V_{k}$ is the vessel velocity. The discretization interval Δt can be set according to the simulation resolution required. Equation (18) constitute a discrete position model $L_{i}$ for the *i*-th vessel,

(19) $$x_{i}\left(k+1\right)=L_{i}\left(u_{i},\;x_{i}\left(k\right)\right),$$

with input vector $u_{i}=\left[\begin{array}{cc} a & V \end{array}\right]\;$ and state vector $x_{i}=\left[\begin{array}{ccc} \theta & x & y \end{array}\right]$. By evaluating the discrete vessel position model $L_{i}$ for {1,2,…,n} consecutive timesteps, where *n* the total timesteps, a trajectory $T_{i}$ can be created for the i-th vessel, as shown in Equation (13).

### 3.2. Collision Avoidance with Mpc and Obstacle Trajectory Prediction Models

The MPC framework has demonstrated its aptitude in handling the uncertainties and nonlinearities of the collision avoidance problem multiple times in the literature [9,49]; however, no other works have incorporated a nonlinear data-driven obstacle trajectory prediction model in their formulation. In MPC, the optimal moves of the controlled vessels are calculated for multiple steps ahead by solving a constrained optimization problem, with constraints in real time, for each controller sample time $t_{cst}$. The cost function of the optimization problem is constituted by two horizons, namely the prediction horizon $h_{p}$ and the control horizon $h_{c}$; the first accounts for the total discrete timesteps ahead that the model can be evaluated, while the second for the number of timesteps that the control variables can be modified. Given a set of controlled vessels $V_{c}=\left\{1,\;2,\ldots ,N_{c}\right\}$ and a set of non-controlled or obstacle vessels $V_{o}=\left\{1,\;2,\ldots ,N_{o}\right\}$ where $N_{c}$ and $N_{o}$ are the total number of controlled and non-controlled vessels, respectively, the optimization problem’s cost function can be formulated as the summation of all the cost functions of the respective controlled vessels for the *k*th timestep:

(20) $$\underset{U\left(k\right)}{\min}\sum_{i\in V}C_{i}\left(X_{c}\left(k\right),X_{o}\left(k\right)\right)\;s.t.\;U_{u}\leq U\left(k\right)\leq U_{l}\;N\left(X_{c}\left(k\right),X_{o}\left(k\right)\right)\geq d_{e}\;P\left(X_{c}\left(k\right),X_{o}\left(k\right)\right)=0$$

Here, U(k) is the input matrix and is created by the horizontal concatenation of the input vectors of all controlled vessels $V_{c}$, up until the control horizon $h_{c}$:

(21) $$U\left(k\right)=\left[\begin{array}{ccc} u_{1}\left(k\right) & \begin{array}{cc} u_{1}\left(k+1\right) & \cdots \end{array} & u_{1}\left(k+h_{c}-1\right)\\ \vdots & \ddots & \vdots \\ u_{N_{c}}\left(k\right) & \begin{array}{cc} u_{N_{c}}\left(k+1\right) & \cdots \end{array} & u_{N_{c}}\left(k+h_{c}-1\right) \end{array}\right].$$

Next $X_{c}\left(k\right)$ and $X_{o}\left(k\right)$ are the controlled and non-controlled vessel state matrices, respectively, and are created by the horizontal concatenation of the state vectors of all controlled and non-controlled vessels $V_{c}$ and $V_{o}$, respectively, up to the prediction horizon $h_{p}$.

(22) $$\begin{array}{c} X_{c}\left(k\right)=\left[\begin{array}{ccc} x_{c,1}\left(k\right) & \begin{array}{cc} x_{c,1}\left(k+1\right) & \cdots \end{array} & x_{c,1}\left(k+h_{p}-1\right)\\ \vdots & \ddots & \vdots \\ x_{c,N_{c}}\left(k\right) & \begin{array}{cc} x_{c,N_{c}}\left(k+1\right) & \cdots \end{array} & x_{c,N_{c}}\left(k+h_{p}-1\right) \end{array}\right]\\ X_{o}\left(k\right)=\left[\begin{array}{ccc} x_{o,1}\left(k\right) & \begin{array}{cc} x_{o,1}\left(k+1\right) & \cdots \end{array} & x_{o,1}\left(k+h_{p}-1\right)\\ \vdots & \ddots & \vdots \\ x_{o,N_{o}}\left(k\right) & \begin{array}{cc} x_{o,N_{o}}\left(k+1\right) & \cdots \end{array} & x_{o,N_{o}}\left(k+h_{p}-1\right) \end{array}\right] \end{array}$$

For simplicity, because consecutive states $x_{i}\left(k\right)$ up to $x_{i}\left(k+h_{p}-1\right)$ constitute a single trajectory $T_{i}\left(k\right)$, one can write $X_{c}\left(k\right)$ and $X_{o}\left(k\right)$ as the concatenation of the trajectories of the respective vessel sets $V_{c}$, $V_{o}$ as per Equation (13):

(23) $$X_{c}\left(k\right)=\left[\begin{array}{c} T_{c,1}\left(k\right)\\ \vdots \\ T_{c,\;N_{c}}\left(k\right) \end{array}\right]\;\;\;X_{o}\left(k\right)=\left[\begin{array}{c} T_{o,1}\left(k\right)\\ \vdots \\ T_{o,N_{o}}\left(k\right) \end{array}\right].$$

Next, $C_{i}\left(k\right)$ is the cost function of the *i*-th controlled vessel, formulated as follows:

(24) $$C_{i}\left(k\right)=F_{i}\left(X\left(k\right)\right)+a_{G}G^{2}\left(U_{i}\left(k\right)\right)$$

Here, X(k) is the vertical concatenation of the two state matrices $X_{c}\left(k\right)$, $\;X_{o}\left(k\right)$, containing the trajectories of all vessels $\;V=V_{c}⋃V_{o}$

(25) $$X\left(k\right)=\left[\begin{array}{ccc} T_{c,1}\left(k\right) & \ldots & \begin{array}{ccc} T_{c,\;N_{c}}\left(k\right) & T_{o,1}\left(k\right) & \begin{array}{cc} \ldots & T_{o,N_{o}}\left(k\right) \end{array} \end{array} \end{array}\right]\prime .$$

The cost function is comprised by two terms $F_{i}$ and G, each concerned with the prediction and control horizon, respectively. The presence of G term, weighted by the $a_{G}$ parameter, encourages the smoothness of the control actions and, consequently, the generated trajectories of the controlled vessels

(26) $$G\left(U_{i}\left(k\right)\right)=\overset{h_{c}-1}{\underset{j=1}{\sum }}\|U_{i,j+1}\left(k\right)-U_{i,j}\left(k\right)\|.$$

Term $F_{i}$ consolidates the collision avoidance and course keeping objectives, and is specific to the *i*-th vessel

(27) $$F_{i}\left(X\left(k\right)\right)=a_{r}\sum_{j\in V\backslash i}\left(f_{r,ij}{}^{2}\left(X_{i}\left(k\right),X_{j}\left(k\right)\right)\right)\;\frac{1}{\left|V\backslash i\right|}+a_{d}\;f_{d,i}{}^{2}\left(X_{i}\left(k\right)\right).$$

In Equation (26), $f_{r,ij}$ is the collision risk between the *i*-th and the *j*-th vessel, as calculated using their respective trajectories $X_{i}\left(k\right),\;X_{j}\left(k\right)$ by applying Equation (12), and $f_{d,i}$ is the deviation from the original trajectory $T_{OG,\;i}$, as expressed in Equation (14). Both terms are weighted by the $a_{r}$ and $a_{d}$ weighting parameters, respectively. Since we are concerned with the safety of the generated trajectory throughout the whole prediction horizon, the mean collision risk from all vessels in V\i is evaluated, in contrast to other approaches [5], where only the maximum collision risk at time *k* is minimized. This way, all possible collision risks are addressed and reduced simultaneously, thus avoiding the adverse possibility of evading one collision risk and increasing another. Moreover, the reason that risk avoidance is used as a control objective in Equation (27) and not as a hard optimization constraint is to ensure that the MPC optimization problem of Equation (20) will not fail in the case of the existence of an inescapable collision risk; as shown in Equation (12), risk is a function of distance to CPA, meaning that the controller will continue to attempt to maximize that distance, thus fulfilling the control intention in such an encounter. However, in order to guarantee that collisions will be avoided, one more constraint to the MPC optimization problem is added by setting an emergency distance $d_{e}$ (where $d_{e}<d_{min})$; to be more specific, the vector N contains the DCPAs of all controlled vessels $V_{c}$, which are required to be above the emergency distance.

At this point, it must be noted that since the state matrix X(k) consolidates all vessel trajectories, controlled and non-controlled alike, a degree of cooperation is induced between the respective controlled vessels $V_{c}$. Lastly, returning to the optimization problem denoted in Equation (20), the U(k) input matrix is bounded by the upper and lower matrices $U_{u}$**,** $U_{l}$, respectively. The vector P contains the COLREG non-compliance penalties for the controlled vessels $V_{c}$ as calculated in Equation (15), which are required to be zero via an equality constraint.

The next item to be addressed regarding the MPC formulation is the used model that maps the input variables U to the state variables of the controlled vessels $X_{c}$. Here, the 1st order linear Nomoto model is used, as described in Equation (18), with the addition of input noise that accounts for modeling error *e* and environmental parameters:

(28) $$x_{c,i}\left(k+1\right)={\hat{L}}_{i}\left(u_{i},\;x_{c,i}\left(k\right)\right),\;\;where\;i\in V_{c}\;{\hat{L}}_{i}\left(u_{i},x_{c,i}\left(k\right)\right)=L_{i}\left(u_{i}+e\left(u_{i}\right),\;x_{c,i}\left(k\right)\right),where\;e\left(u_{i}\right)=u_{i}\;G\left(0,\sigma^{2}\right).$$

Here, G is a random variable sampled from a Gaussian distribution with a standard deviation of *σ*. Finally, the state matrix of the non-controlled vessels $X_{o}\left(k\right)$ is assumed to be unknown for the scope of this research, and thus, an estimation is required, based on past positions. For this task, the RBF prediction model presented in Section 2.3 is employed for each non-controlled vessel *j*, and its trajectory for the *k*-th timestep is estimated using its past nine positions:

(29) $${\hat{T}}_{o,j}\left(k\right)=RBF\left(x_{o,j}\left(k\right),x_{o,j}\left(k-1\right),\ldots ,x_{o,j}\left(k-9\right)\right),\;\;where\;j\in V_{o}.$$

Next, in order to alleviate a possible computational burden for the MPC optimization problem, an important assumption should be made. The formulation of the control scheme as-presented would give rise to a high-dimensional search space for the MPC optimization problem, thus greatly hindering its effective solution. It is assumed then that all vessels retain their initial speed, with the only controllable variable being the vessel’s rudder angle; this way, the total number of control variables is reduced by half. This approach to the collision avoidance problem has occurred in the literature [5], and is not simplistic for two reasons: first, good seafaring practice dictates that course change maneuvers are preferred over speed ones, not only because they conserve energy, but also because they better emphasize the intentions of the vessel to outside observers, such as other vessels in the vicinity. Second, since large container ships will be examined in the scope this case study, their large longitudinal inertia [48] confirms the assumption that the speed remains almost constant during the timeframe of a typical collision avoidance maneuvering scenario. Therefore, for the scope of this paper, the input matrix U at timestep *k* is formulated as follows:

(30) $$U\left(k\right)=\left[\begin{array}{ccc} a_{1}\left(k\right) & \begin{array}{cc} a_{1}\left(k+1\right) & \cdots \end{array} & a_{1}\left(k+h_{c}-1\right)\\ \vdots & \ddots & \vdots \\ a_{N_{c}}\left(k\right) & \begin{array}{cc} a_{N_{c}}\left(k+1\right) & \cdots \end{array} & a_{N_{c}}\left(k+h_{c}-1\right) \end{array}\right],$$

where $a_{i}\left(k\right)$ is the rudder angle of vessel *i* at timestep *k.*

Having defined all aspects of the MPC optimization problem, a reiteration of the challenges of the collision avoidance control problem and how they are addressed by the controller is in order: firstly, the goal of the control design is to generate trajectories for the controlled vessels that are risk-free (Equation (12)), smooth (Equation (26)), COLREG-compliant (Equation (15)), and do not deviate from the original course (Equation (14)). Possible collision risks are assessed by utilizing trajectory predictions for non-controlled (obstacle) vessels in the vicinity. The controllable variables are the rudder angles of the vessels (vessel speed is considered constant), while a discrete 1st order Nomoto model (Equation (28)) is used for the modeling of the vessel dynamics, which was also infused with a noise signal for the purpose of accounting for uncertainties and environmental factors. The aforementioned vessel dynamics model has been compared to its higher-order nonlinear counterparts in [50], and it was shown that vessel course inaccuracies occur only for high yaw rates. Given the fact that the proposed collision avoidance method is concerned with large vessels with slow dynamics, the used vessel dynamics model is deemed adequate for the case. In addition, MPC has shown to be robust against model uncertainties or input noise [36]. Finally, the constraints that must be adhered to when searching for the optimal solution (Equation (20)) are the technical bounds on the controlled variables (i.e., maximum and minimum rudder angles) and the COLREG compliance of the result trajectory.

### 3.3. Control Framework

**Figure 5:**
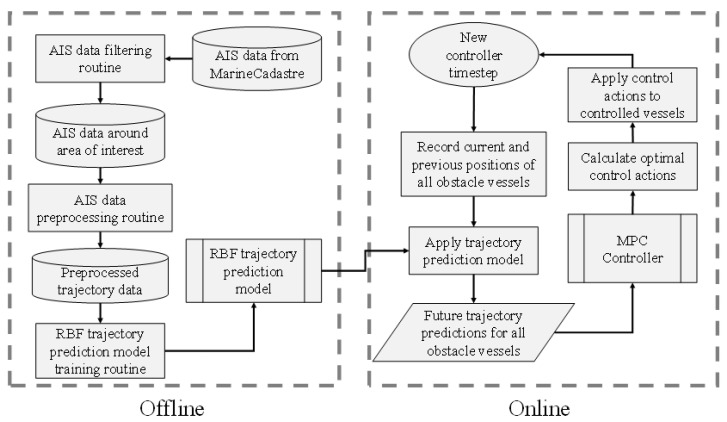
The proposed control framework.

**Figure 6:**
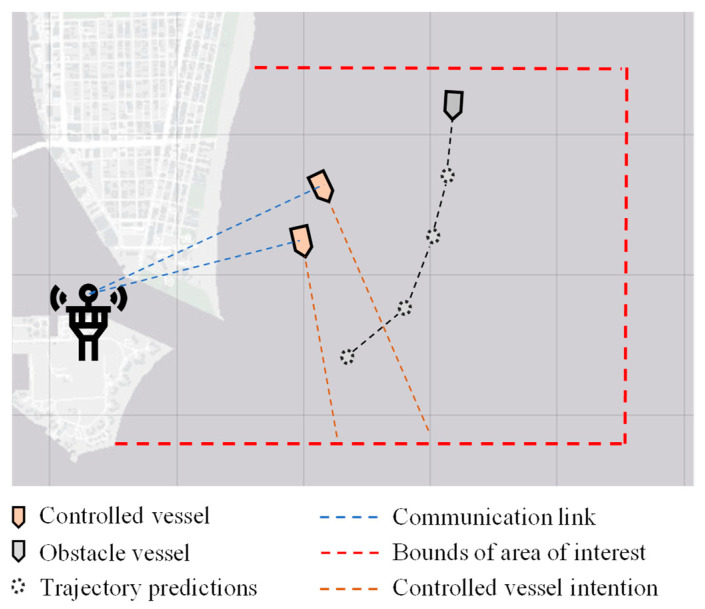
Communications within the proposed control framework.

Having presented the proposed MPC controller, this section describes its integration within a general control framework. As shown in Figure 5, the framework is comprised by an offline and an online process. The offline process corresponds to the RBF trajectory prediction model training, using data from a specific area of interest (for example, a port)—naturally then, it could be undertaken by the port authority. The online process corresponds to the real-time control of autonomous vessels in the presence of obstacle vessels in the area of interest. The MPC collision avoidance controller, as described in Section 3.2, is integrated here and is supplied with real time trajectory predictions of all obstacle vessels in order to calculate the optimal control actions for the controlled vessels. Since the RBF trajectory prediction model has been trained offline in the port authority premises, it is sensible to place the MPC controller there too, and to communicate the computed control actions per control timestep via a communications link with the controlled vessels. Figure 6 demonstrates this concept.

The MPC optimization problem described in Equation (20) is solved using the sequential quadratic programming (SQP) algorithm, which involves iterative calls to the objective function [35]. As shown in Figure 5, the integration of the MPC controller in the control framework requires the calculation of the obstacle vessel trajectory predictions for every controller timestep. Therefore, two main sources of computational complexity arise: the first is the evaluation of the RBF trajectory prediction model, which is shown to be in the order of magnitude of milliseconds [31], meaning that multiple obstacle vessels can be accounted for by the control scheme. The second is the solution of the optimization problem (Equation (20)) by the SQP algorithm, which is known to converge quickly and with few objective function calls [51]. It is concluded that a typical controller timestep duration, comprised by the two aforementioned sources, will not exceed the order of magnitude of seconds, which is considered reasonable given the slow dynamics of large vessels.

## 4. Case Study

In this section, the performance of the proposed multi-ship MPC controller is assessed using real-life obstacle ship trajectories, which were sourced and preprocessed as described in Section 2.4. In order to underline the importance of using sophisticated trajectory prediction models in the context of collision avoidance controller design, the proposed method is compared to an MPC controller that uses straight-line predictions for the trajectories of obstacle ships based on their current course and speed [9]. To this end, two crossing scenarios are examined, while performance indicators of the generated trajectories are extracted and discussed in detail. The simulations were coded and executed on Matlab 2020b, on a computer with an Intel i7 processor and 16 GB RAM. The simulation sample time is 30“. Lastly, the tuning and parameters of the methods are shown in Table 2, while the vessel parameters are shown in Table 3.

### 4.1. Multi-Ship Collision Avoidance Control for the Miami Port

For this case study, two controllable vessels are chosen, moving in parallel to each other and encountering an obstacle vessel moving into the port of Miami. For the performance evaluation of the two controllers, two scenarios are created; the first contains a head-on encounter type, while the second an overtaking maneuver that changes into a crossing encounter as time progresses. In the first scenario, the two controlled vessels are leaving the port of Miami at a course of 110°, when they encounter a single obstacle on their starboard side, which, in turn, is looking to enter the port. In the second scenario, the two controlled vessels are overtaking an obstacle vessel on her port side when, suddenly, she turns port-side in order to enter the port of Miami, crossing into their intended path. The challenge posed by the two scenarios is that the two controllable vessels should maintain a safe distance between each other and the obstacle vessel, while also navigating smoothly and without unnecessary deviation from their original course. It should also be noted that the obstacle vessel is non-controllable and, therefore, follows a predetermined path, without considering other vessels.

**Figure 7:**
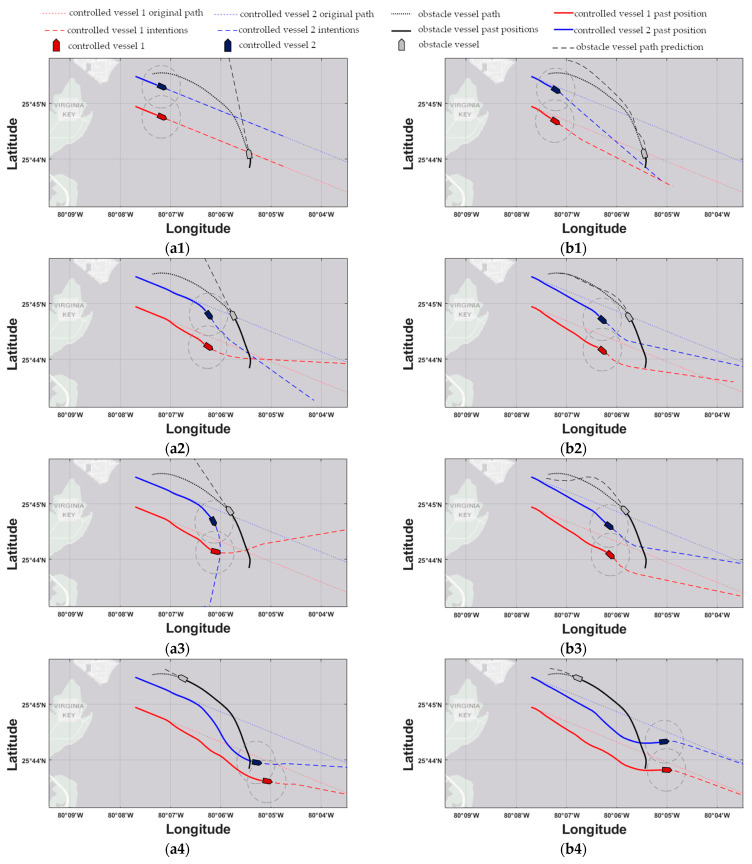
Scenario 1. The left subfigure column refers to the MPC-SLP scheme, while the right to the MPC-RBFP scheme. Subfigures (**a1**,**b1**) refer to time instance 3′, (**a2**,**b2**) to 9′, (**a3**,**b3**) to 10′, and finally, (**a4**,**b4**) to 16.5′.

**Figure 8:**
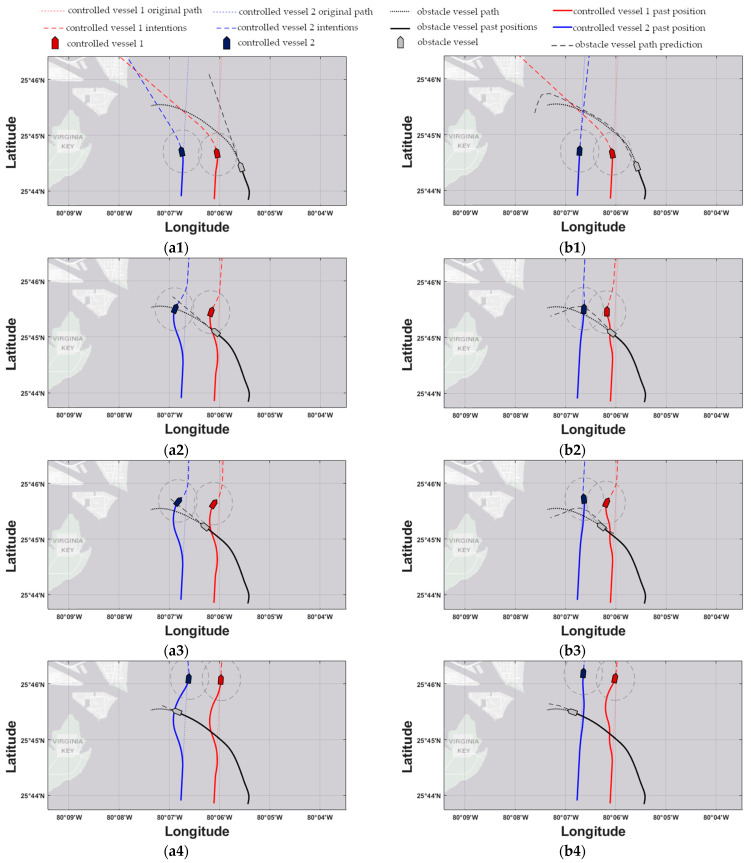
Scenario 2. The left subfigure column refers to the MPC-SLP scheme, while the right to the MPC-RBFP scheme. Subfigures (**a1**,**b1**) refer to time instance 6′, (**a2**,**b2**) to 12′, (**a3**,**b3**) to 13.5′, and finally, (**a4**,**b4**) to 17′.

The response of the MPC controller utilizing straight-line prediction models (hereby referred to as ‘MPC-SLP’) for the first scenario is shown in the left column of the subfigures within Figure 7 for the 3-, 9-, 10-, and 16.5-min timesteps. The response of the proposed MPC controller utilizing RBF prediction models (hereby referred to as ‘MPC-RBFP’) for the same scenario and same time instances are shown in the right column of the subfigures within Figure 8. Next, the responses of MPC-SLP and MPC-RBFP for the second scenario are shown in the left and right subfigure columns of Figure 8, respectively, for the 6-, 12-, 13.5-, and 17-min timesteps. In the aforementioned response figures, the red and blue dotted lines denote the original, undisturbed trajectory for controlled vessels 1 & 2, respectively, while the black dotted line shows the predetermined path that the obstacle ship will follow as the simulation progresses. Next, the red and blue dashed lines denote the trajectory that the controlled vessels intend to follow, as calculated by the current MPC iteration, while the black dashed line shows the current trajectory prediction of the obstacle ship, as utilized by the MPC controller. The grey dashed circles have a radius of $d_{min}$ and denote the safe ship domain for the two controlled vessels; should any vessel enter another’s domain at any time, a collision risk arises. Lastly, the red-colored and blue-colored rectangles mark the controlled vessels 1 & 2 positions, respectively, while the grey rectangle marks the obstacle ship’s position; it should be noted that the markers are not to-scale with the real dimensions of the vessels, since they have been enlarged for graphical convenience.

### 4.2. Discussion

**Figure 9:**
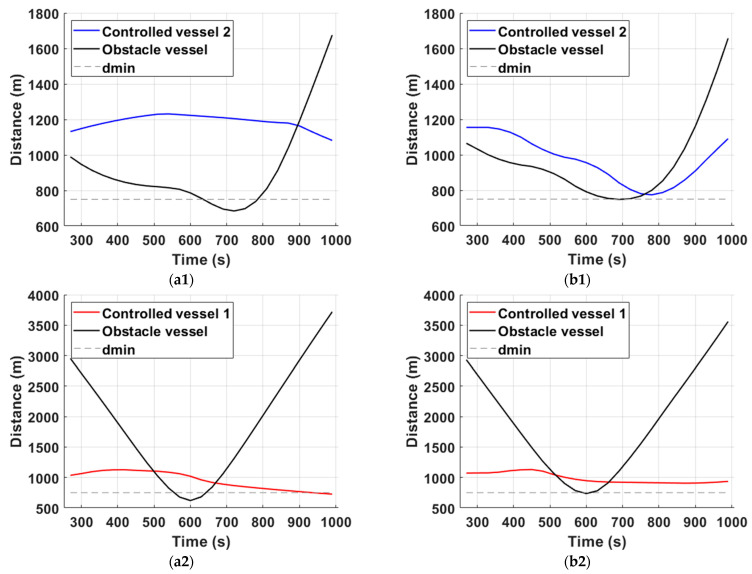
Distance plots for scenarios 1 & 2. The left subfigure column refers to the MPC-SLP scheme, while the second to the MPC-RBFP scheme. Note that for scenario 1 (**a1**,**b1**) and for scenario 2 (**a2**,**b2**), the MPC-SLP violates the lower limit on distance from CPA; therefore, its trajectories are deemed unsafe.

Firstly, in order to assist the discussion in this subsection, distance plots are generated for the controlled vessels that are in closest proximity with the obstacle ship for each scenario (see Figure 9). In addition, the performance metrics for each controller in each scenario are shown in Table 4.

For the head-on encounter of scenario 1, the correct trajectory prediction of the obstacle ship proves vital for the success of the proposed scheme. Considering timestep 3 (see Figure 7(a1,b1)), the MPC-RBFP scheme is already applying evasive control actions, since the correct inference of the general direction of the obstacle ship has given rise to a possible collision risk in the near future. In contrast, the MPC-SLP controller does not apply any control actions yet, because, based on the straight-line prediction model that it utilizes, the obstacle vessel will continue north and, thus, remain well clear of the controlled vessels. For the same reason, it takes MPC-SLP another 5′ minutes in order to correctly assess the collision risk and apply decisive control actions, but by then it is too late; by timestep 9′ (see Figure 7(a3,b3)), controlled vessel 2 reaches its CPA with the obstacle ship, with a DCPA of 680 m for controlled vessel 2, well below the acceptable minimum distance $d_{min}$, as shown in Figure 9(a1). In contrast, the MPC-RBFP controller generates a smooth, safe, and consistent trajectory, owed to the correct trajectory prediction of the obstacle vessel. Not only does it reach an acceptable DCPA of 751 m for controlled vessel 2, but it also manages to apply consistent control actions and not significantly deviate from the original course, as shown in Table 4**.**

Next, the performance of the two controllers is assessed in an overtaking/crossing encounter in scenario 2. Here, the effect of the used trajectory prediction models is once again eminent: At timestep 6 (see Figure 8(a1,b1)), MPC-RBFP calculates a sharp control move to port-side for controlled vessel 1 in anticipation of the obstacle ship’s crossing towards the port of Miami; in contrast, MPC-SLP applies a lower rate of steering for controlled vessel 1, because the straight-line trajectory prediction places its CPA with the obstacle ship at a later time instance. This failure to correctly place the CPAs has adverse effects on vessel 2 trajectory too, since it is displaced unnecessarily to the left in false anticipation of a collision risk. In addition, the obstacle ship crosses into the domain of controlled vessel 1 (see Figure 8(a2)) once it changes course towards the Miami port at timestep 8′. On the other hand, the MPC-RBFP scheme places controlled vessel 1 in a better position to narrowly evade the breach of its safe domain (see Figure 8(b2)) throughout the simulation. This performance is owed to the trajectory that the RBF model generated for the obstacle vessel, placing its predicted CPA much closer to the real CPA for both controlled vessels. Moreover, it should be noted that for scenario 2, unnecessary deviations from the original course are avoided for controlled vessel 2, as indicated by the total deviation values in Table 4. In general, the proposed method achieves a lower overall cost for the generated trajectories, as shown in Table 4, while obtaining a certain degree of cooperation between the two vessels, where one makes way for the other in anticipation of their upcoming evasive maneuvers. Moreover, the results show that the proposed method exhibits robust characteristics against environmental effects, which are modeled as input noise in the vessel dynamic model for the scope of the simulations, while accounting for COLREGs. Lastly, the average CPU time evaluation of the MPC calculation was recorded as 7s for both scenarios, which is well within the allocated simulation controller timestep $t_{cst}$ of 60 s, proving, in fact, that the proposed method is scalable to a greater number of controlled and obstacle vessels.

## 5. Conclusions

In this paper, a multi-ship MPC controller utilizing RBF obstacle ship trajectory prediction models trained on real AIS data is proposed for the collision avoidance task in busy ports or waterways. The proposed method is compared to an MPC controller using straight-line obstacle ship trajectory prediction models for a real simulation case for the port of Miami. The simulations have shown that the incorporation of a trajectory prediction model with a moderate degree of accuracy greatly benefits the performance of a collision avoidance controller; this is due to the fact that a collision risk can be detected earlier and in time, so that it can be accommodated by the slow maneuvering dynamics of larger vessels such as container ships. Moreover, this early detection enables the planning of a more economic trajectory for the controlled vessels and enables their better cooperation.

## Figures and Tables

**Table 1:** Performance metrics of the produced RBF NN model.

	RBF NN	
	Latitude (*y*)	Longitude (*x*)
Mean RMSE (deg)	1439∙10^−6^	1567∙10^−6^
	Best combined RBF models	
Mean RMSHFD (m)	1200	

**Table 2:** MPC tuning parameters.

Parameter	Description	Value
$t_{cst}$	Controller sample time	1′
$h_{p}$	Control horizon	5
$h_{c}$	Prediction horizon	15
$a_{0}$	Risk function scaling parameter	3
$a_{r}$	Risk term weighting parameter	1
$a_{d}$	Course deviation term weighting parameter	0.05
$a_{G}$	Control action smoothness term weighting parameter	5

**Table 3:** Vessel parameters.

Parameter	Description	Value
$d_{min}$	Minimum allowable DCPA for risk calculation	750 m
$d_{e}$	Emergency distance	200 m
$t_{min}$	Minimum allowable TCPA for risk calculation	10′
$K_{s}$	Rudder gain constant	0.5
$T_{s}$	Rudder time constant	2

**Table 4:** Performance metrics for the generated trajectories of the MPC-RBFP and MPC-SLP schemes for the two simulation scenarios.

		Scenario 1		Scenario 2	
s	Controlled Vessel	MPC-RBFP	MPC-SLP	MPC-RBFP	MPC-SLP
**Course** **deviations** ^(1)^	1	1.31·10^4^	2.21·10^4^	0.658·10^4^	0.521·10^4^
	2	1.49·10^4^	2.85·10^4^	0.404·10^4^	0.529·10^4^
**Control action smoothness** ^(2)^	1	307.35	476.59	242.12	167.85
	2	290.94	424.43	92.47	128.41
**Risk of trajectory** ^(3)^	1	0	4.032·10^6^	0	0
	2	0	0	0	3.949·10^6^
**Cost of trajectory** ^(4)^	1	9.05·10^6^	1.62·10^13^	2.63·10^6^	1.49·10^6^
	2	2.63·10^6^	4.15·10^7^	8.58·10^5^	1.55·10^13^

^(1)^ As calculated by Equation (14). ^(2)^ As calculated by Equation (26). ^(3)^ As calculated by Equation (12). ^(4)^ As calculated by Equation (24).

## Data Availability

In this work, the publicly available AIS dataset provided by the Marine Cadastre service (www.marinecadastre.gov, accessed on 25 July 2021) has been used for trajectory modeling purposes.
